# Supplementary material for: Single-cell profiling of lncRNA expression during Ebola virus infection in rhesus macaques
Source: Nat Commun. 2023 Jun 30;14:3866. doi: 10.1038/s41467-023-39627-7 (PMC10313701; doi:10.1038/s41467-023-39627-7)
Supplement: Supplementary file 1 — Supplementary Information [file 41467_2023_39627_MOESM1_ESM.pdf]

Supplementary Information

Single-cell profiling of lncRNA expression during Ebola virus infection in rhesus macaques

Luisa Santus[#1,2], Maria Sopena-Rios[#1], Raquel García-Pérez[+1,] Aaron E Lin[+3,4,5], Gordon C Adams[3,4], Kayla G Barnes[4,6,7], Katherine J Siddle[3,4], Shirlee Wohl[3,4,8], Ferran Reverter[9], John L Rinn[10], Richard S Bennett[11], Lisa E Hensley[*11], Pardis C Sabeti[*3,4,5,12], Marta Melé[*1]

[1]Life Sciences Department, Barcelona Supercomputing Center, Barcelona, Catalonia 08034, Spain;

[2]Centre for Genomic Regulation (CRG), The Barcelona Institute for Science and Technology, Barcelona, Spain;

[3]FAS Center for Systems Biology, Department of Organismic and Evolutionary Biology, Harvard University, Cambridge, MA 02138, USA;

[4]Broad Institute of MIT and Harvard, Cambridge, MA 02142, USA;

[5]Harvard Program in Virology, Harvard Medical School, Boston, MA 02115, USA;

[6]Department of Immunology and Infectious Diseases, Harvard T.H. Chan School of Public Health, Harvard University, Boston, MA 02115, USA;

[7]Liverpool School of Tropical Medicine, Liverpool UK, L3 5QA;

[8]The Scripps Research Institute, Department of Immunology and Microbiology, La Jolla, CA, USA;

[9]Department of Genetics, Microbiology and Statistics University of Barcelona, Barcelona, Spain;

[10]Department of Biochemistry, University of Colorado Boulder, Boulder 80303, USA;

[11]Integrated Research Facility, Division of Clinical Research, National Institute of Allergy and Infectious Diseases, National Institutes of Health, Frederick, MD 21702, USA;

[12]Howard Hughes Medical Institute, Chevy Chase, MD 20815, USA;

#,+ contributed equally

* corresponding authors: lisa.hensley@nih.gov, pardis@broadinstitute.org, marta.mele.messeguer@gmail.com

Supplementary Figure 1

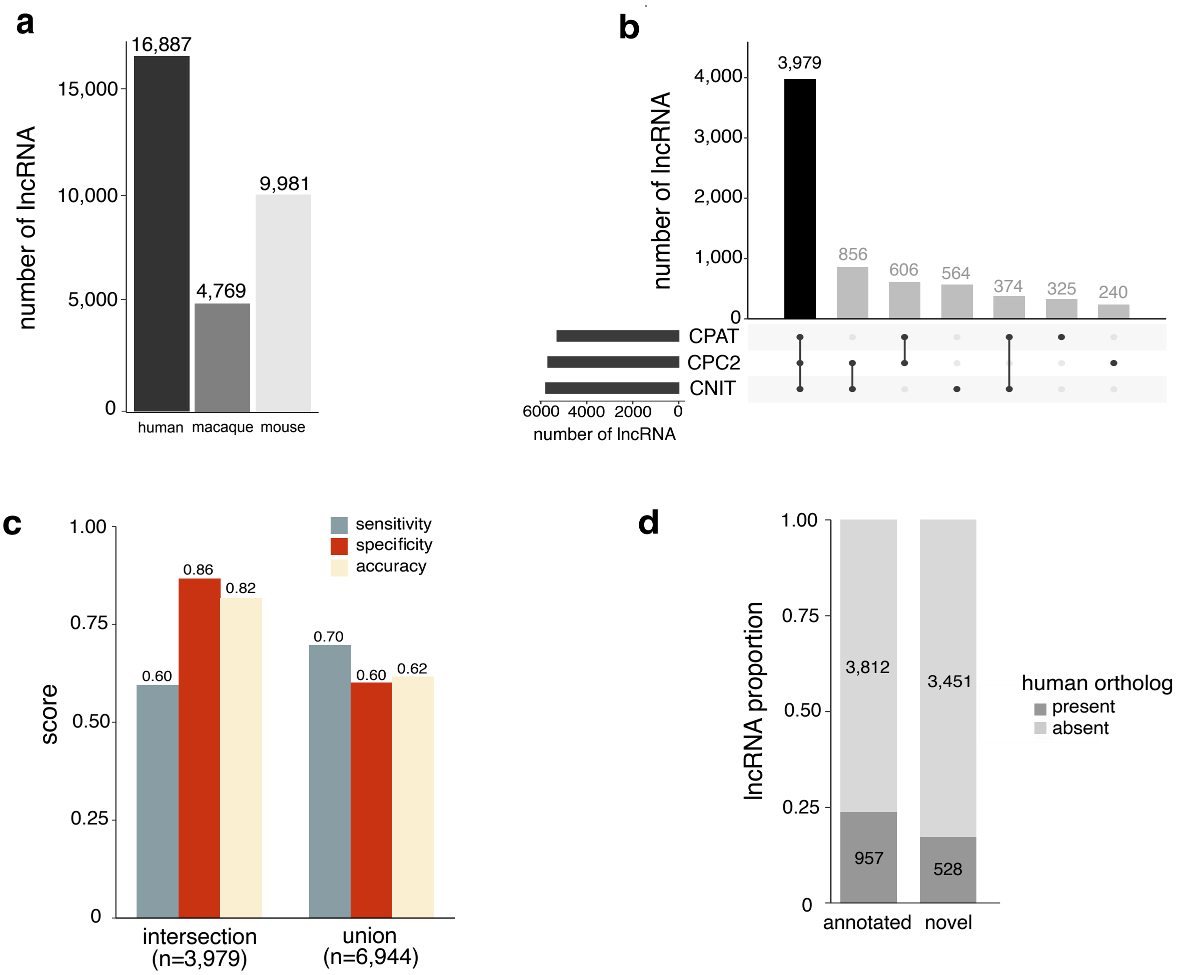

Supplementary Fig. 1. Identification and characterization of novel lncRNAs. (A) Number of annotated lncRNA genes in human, macaque, and mouse in the Ensembl release 100. **(B)** Overlap of the genes predicted as non-coding by the CPAT, CPC2, and CNIT tools. Highlighted in black are the number of genes predicted as non-coding by the three tools used for downstream analyses. **(C)** Benchmarking measures for non-coding genes obtained by the intersection (left) and union (right) of biotype predictions from CPAT, CPC2, and CNIT. **(D)** Proportion of genes for which we identified a human ortholog in annotated (left) and novel lncRNAs (right).

Supplementary Figure 2

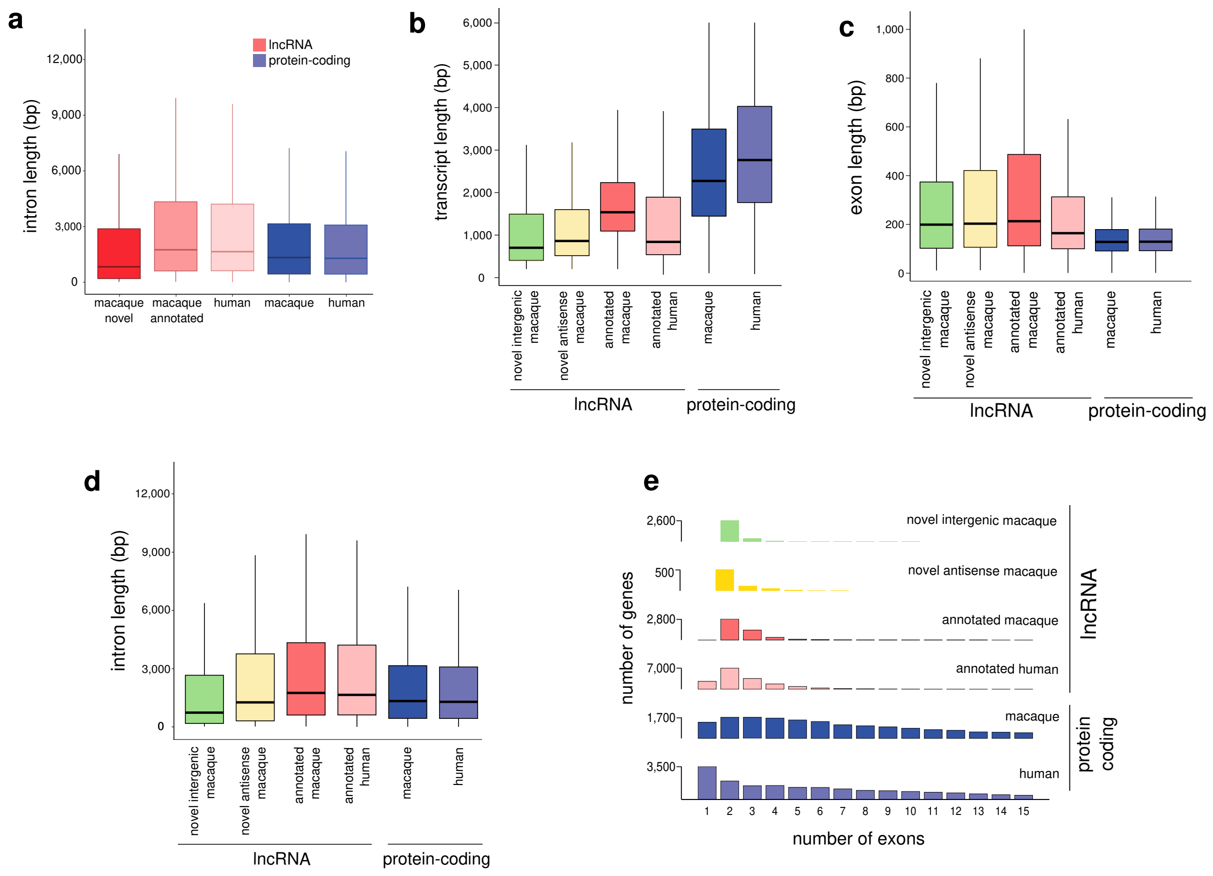

Supplementary Fig. 2. Novel intergenic and novel antisense lncRNAs resemble annotated lncRNAs. (**A**) Distribution of intron length of macaque novel and annotated lncRNA (red) and protein-coding genes (blue). (**B**) Distribution of transcript length, (**C**) exon length (**D**) intron length, and **(E)** number of exons per transcript in novel and annotated macaque and human intergenic and antisense lncRNAs and protein-coding genes. Macaque lncRNAs: novel intergenic (genes: n=3,191, exons: n= 7,225), novel antisense lncRNAs (genes: n=788, exons: n=1,901), annotated (genes: n=4,769, exons: 12,370), human lncRNAs (genes: n=16,887, exons: n=47,020). Macaque protein-coding (genes: n=21,591, exons: n=195,853), human protein-coding (genes: n=199,097, exons: n=199,097). All boxplots display the median and the first and third quartiles (the 25th and 75th percentiles) of the data. The whiskers extend to the highest and lowest values within 1.5 times the interquartile range (IQR) of the data.

Supplementary Figure 3

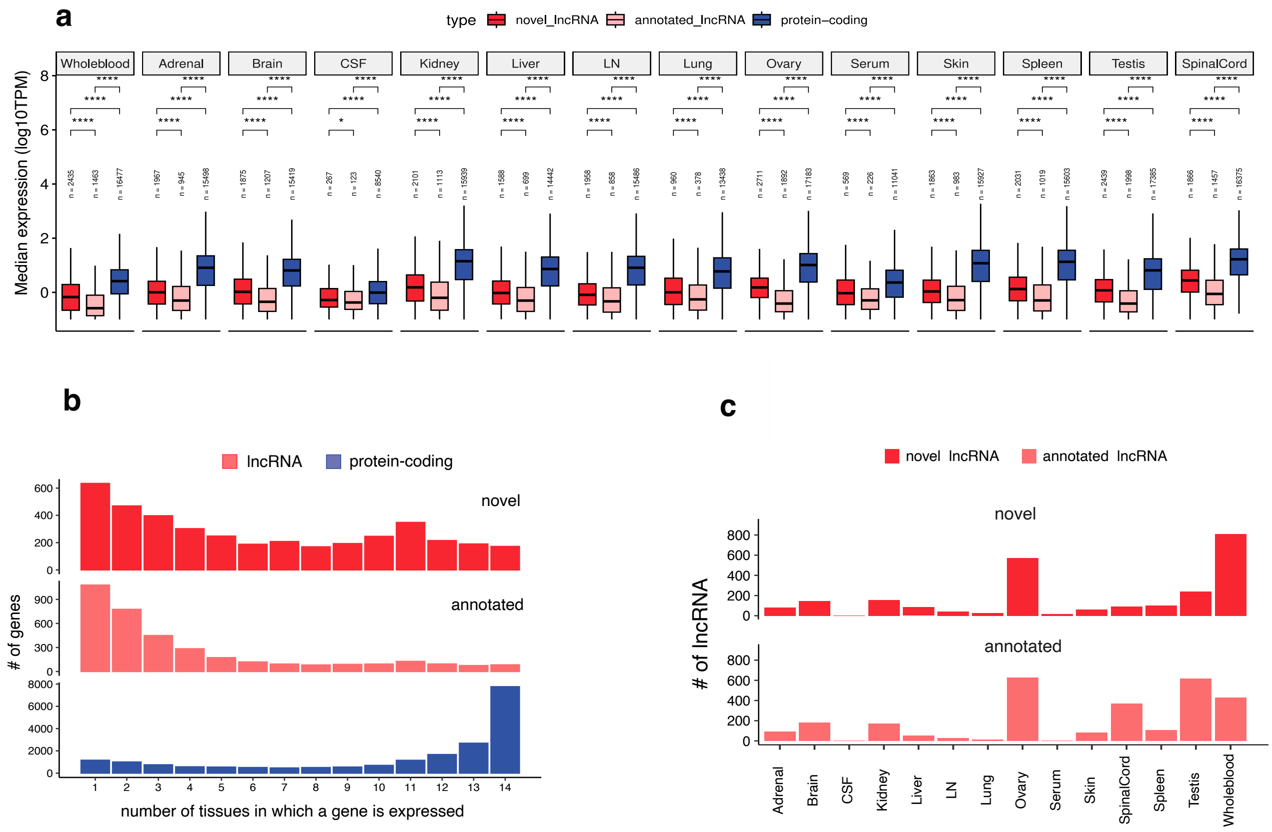

Supplementary Fig. 3. Patterns of expression of novel and annotated lncRNA. (**A**) Distribution of average gene expression per tissue. Mann-Whitney U test, P-values < 2.2×10^{-16}. All boxplots display the median and the first and third quartiles (the 25th and 75th percentiles) of the data. The whiskers extend to the highest and lowest values within 1.5 times the interquartile range (IQR) of the data. (**B**) Number of tissues in which macaque novel and annotated lncRNA (red) and protein-coding genes (blue) genes are expressed. **(C)** Bar plot showing the distribution of novel (top) and annotated (bottom) tissue-specific lncRNAs (Tau > 0.7) across tissues.

Supplementary Figure 4

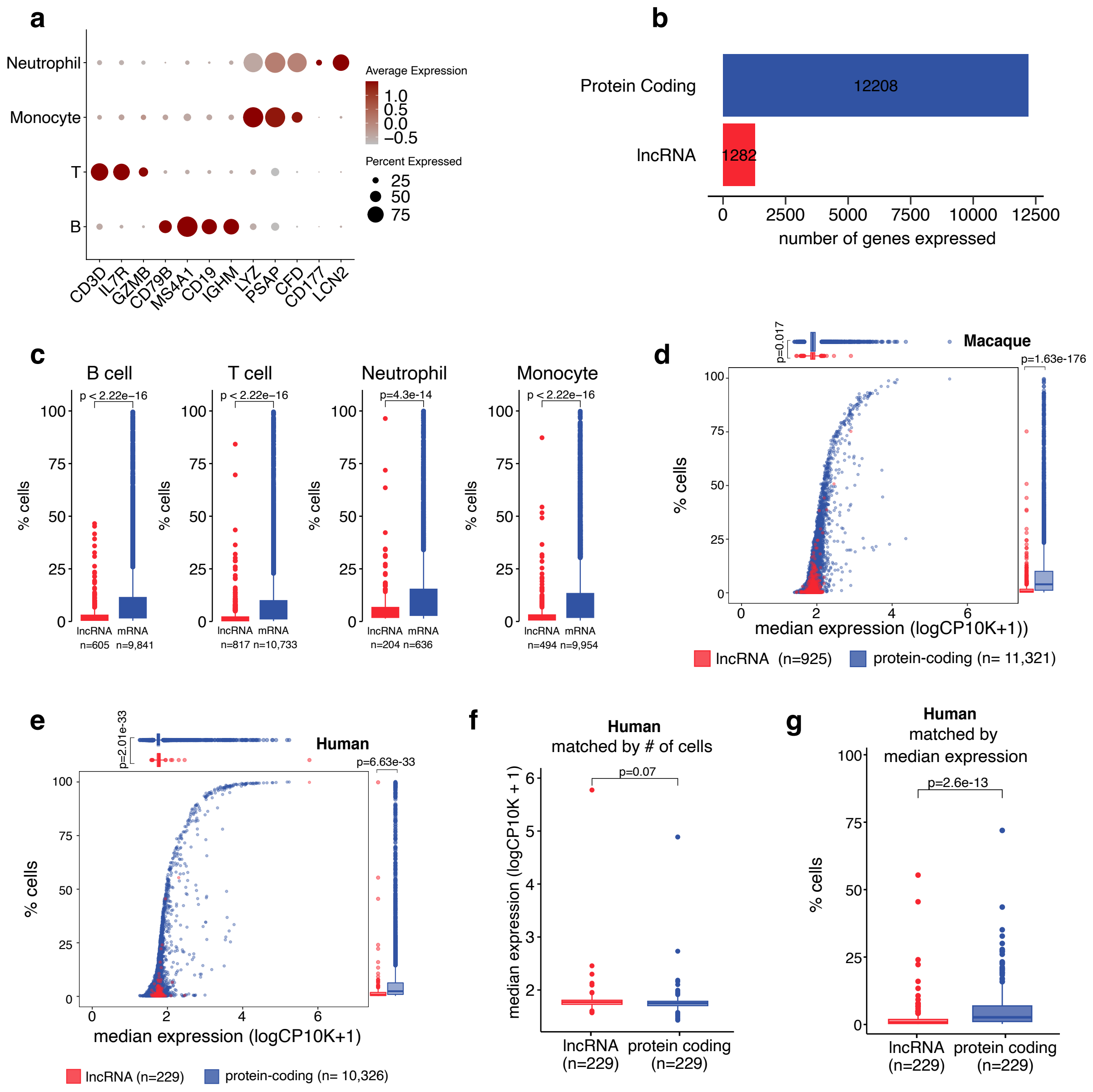

Supplementary Fig. 4. Expression levels and percentage of cells in which lncRNA and protein-coding genes were expressed at single-cell resolution. (A) Dot plot showing the expression of cell-type markers which were used for cell type assignment in the *in vivo* dataset (see Methods). Dots' colors represent the expression level (z-scored log(CP10K)) of the gene in each cell type. Dots' sizes represent the percentage of cells in which the gene is detected as expressed per cell type. **(B)** Number of lncRNA (red) and protein-coding (genes) after the filtering (> 1 logCPK10+1 in more than 10 cells) **(C)** Distribution of the percentage of cells (log10) in which lncRNA and protein-coding genes were expressed per cell type. **(D)** Scatter plot displaying the median expression level of lncRNA (red) and protein-coding genes (blue) versus the percentage of cells in which they are expressed. **(E)** Same as D but for lncRNA and protein-coding genes in human PBMCs. **(F)** Median expression of lncRNAs and protein-coding genes in human PBMCs

matched by the percentage of cells in which they are expressed. **(G)** Percentage of cells in which lncRNA and protein-coding genes are expressed in human PBMCs matched by their median expression levels. Mann-Whitney U test. All boxplots display the median and the first and third quartiles (the 25th and 75th percentiles) of the data. The whiskers extend to the highest and lowest values within 1.5 times the interquartile range (IQR) of the data.

Supplemental Figure 5

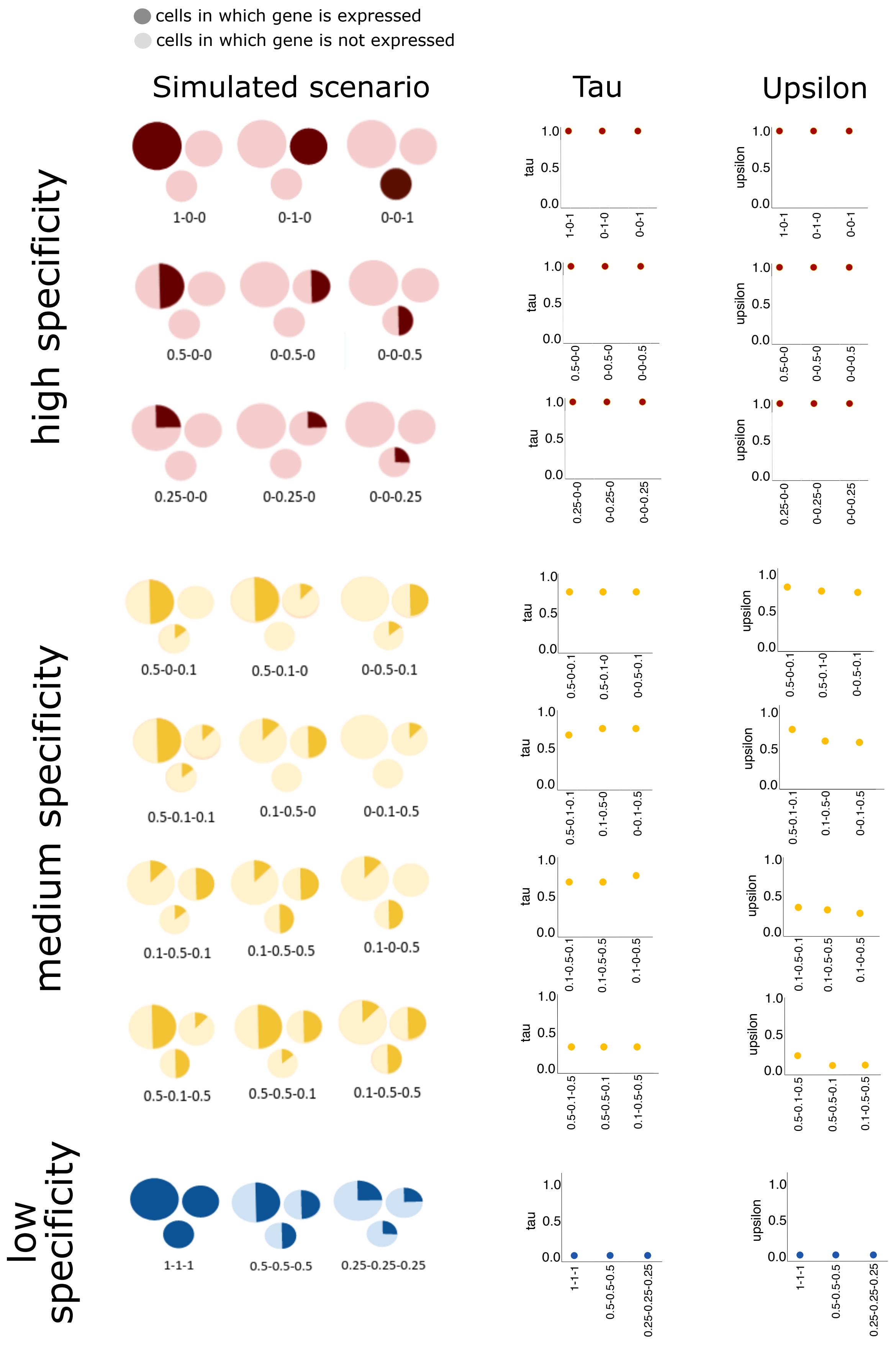

Supplementary Fig. 5. Cell-type specificity scores' behavior across simulated scenarios. Schematic representation of the simulated scenarios for genes with high, intermediate, or low cell-type specificities (red, yellow, and blue respectively). Cells are split into three cell types, which represent 50%, 30%, and 20% of the total number of cells. Proportions highlighted in darker and lighter colors represent cells in which the gene is expressed and not expressed respectively. Numbers below each pie chart correspond to the proportion of cells in which the gene is expressed, sorted from the cell types with the highest proportion to the lowest proportion of cells. High cell-type specific genes can be expressed in 100%, 50%, or 25% of the cells of exclusively one cell type. Genes with intermediate cell-type specificity can be (1) expressed in 50% of the cells of one cell type, in 10% of the cells of a second cell type, and show no expression in the cells of the remaining cell type, (2) expressed in 50% of the cells of one cell-type and in 10% of the cells of all other cell types, or (3) expressed in 50% of the cells of two cell types and not expressed in the cells of the remaining cell-type. Lastly, low cell-type specific genes can be expressed in 100%, 50%, or 10% of the cells of all cell types. Corresponding Tau (left) and Upsilon (right) cell-type specificity scores.

Supplementary Figure 6

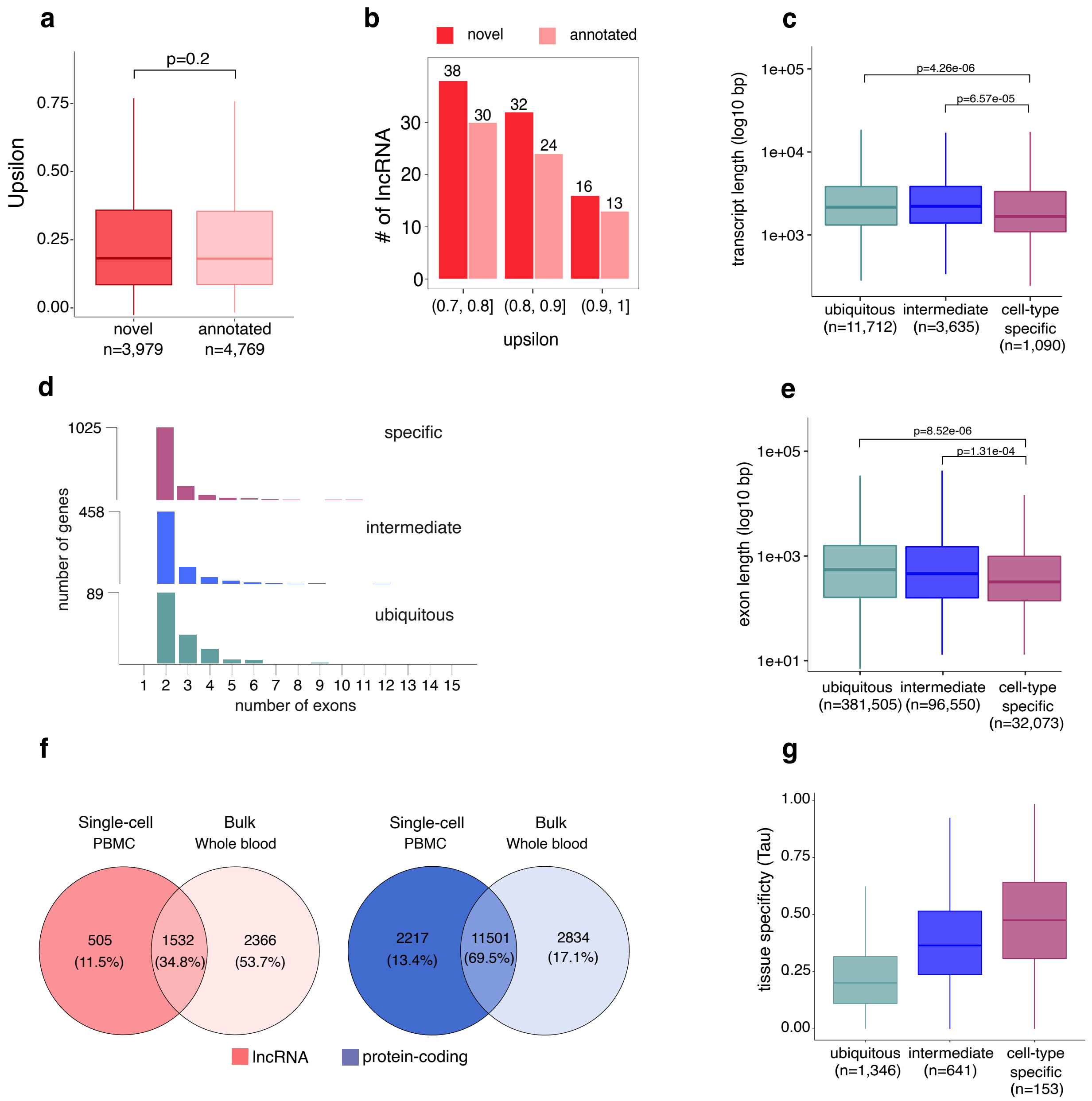

Supplementary Fig. 6. Characterization of cell-type specific lncRNAs **(A)** Distribution of Upsilon specificity scores of novel (red) and annotated (pink) lncRNAs. Mann-Whitney U test. **(B)** Bar plot showing the number of cell-type specific lncRNAs (Upsilon > 0.7), binned by Upsilon ranges. Distribution of transcript length, **(C)** number of exons **(D)** and exon length **(E)** of ubiquitous, intermediate, and cell-type specific lncRNA. Mann-Whitney U test. **(F)** Venn Diagram of the overlap between expressed lncRNA (red) and protein-coding (blue) genes in single-cell PBMC and whole blood bulk RNA-seq datasets. **(G)** Distribution of tissue-specificity Tau scores of ubiquitous, intermediate, and cell-type specific protein-coding genes. All boxplots display the median and the first and third quartiles (the 25th and 75th percentiles) of the data. The whiskers extend to the highest and lowest values within 1.5 times the interquartile range (IQR) of the data.

Supplementary Figure 7

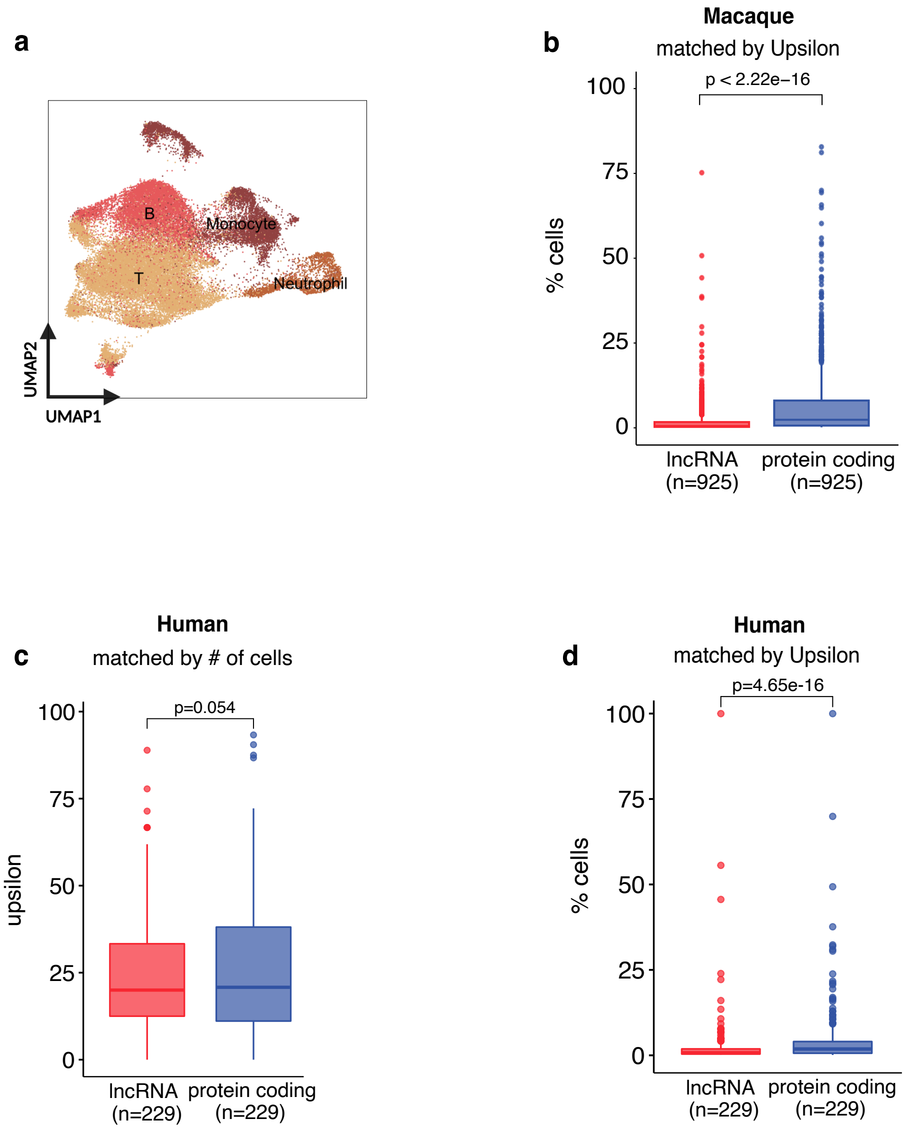

Supplementary Fig. 7. Proportion of cells in which lncRNAs and protein-coding genes were expressed and their cell-type specificity. (A) UMAP embedding of 38,067 cells using lncRNAs expression. Cell types are indicated by the different colors. **(B)** Distribution of percentage of cell expressing lncRNA and protein-coding genes matched by the cell-type specificity score Upsilon. Wilcoxon signed-rank test. **(C)** Upsilon cell-type specificity scores**,** calculated using human healthy PBMCs, of lncRNAs and protein-coding genes matched by the percentage of cells in which they were expressed. Wilcoxon signed-rank test. **(D)** Same human PBMCs dataset but showing the distribution of percentage of cells lncRNAs and protein-coding are expressed when matching by Upsilon. Wilcoxon signed-rank test. All boxplots display the median and the first and third quartiles (the 25th and 75th percentiles) of the data. The whiskers extend to the highest and lowest values within 1.5 times the interquartile range (IQR) of the data.

Supplementary Figure 8

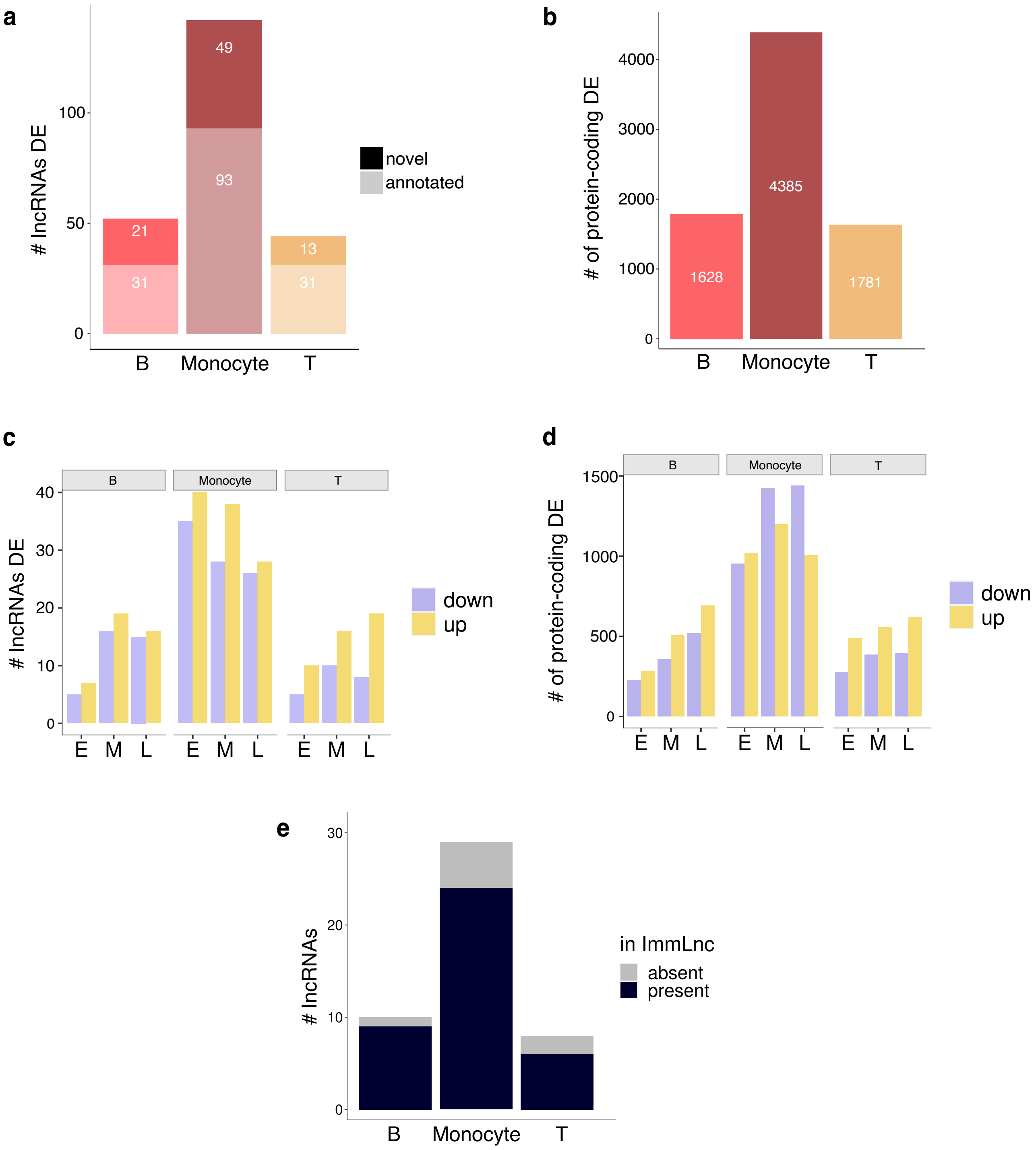

Supplementary Fig. 8. Differential expression patterns of lncRNAs upon EBOV infection. (A) Bar plot showing the number of DE lncRNAs detected upon EBOV infection *in vivo*, colored by cell type and separated by whether they were annotated (lighter colors) or identified through *de novo* annotation (darker colors). **(B)** Bar plot showing the number of DE protein-coding genes detected upon EBOV infection *in vivo* colored by cell type. Bar plot showing the number of DE lncRNAs **(C)** and protein-coding genes **(D)** detected upon EBOV infection *in vivo*, separated by

the directionality of the expression changes. **(E)** Bar plot showing the number of DE lncRNAs detected upon EBOV infection *in vivo* for which we could identify a human ortholog. Color code indicates whether the lncRNAs have been previously reported in ImmLnc

Supplementary Figure 9

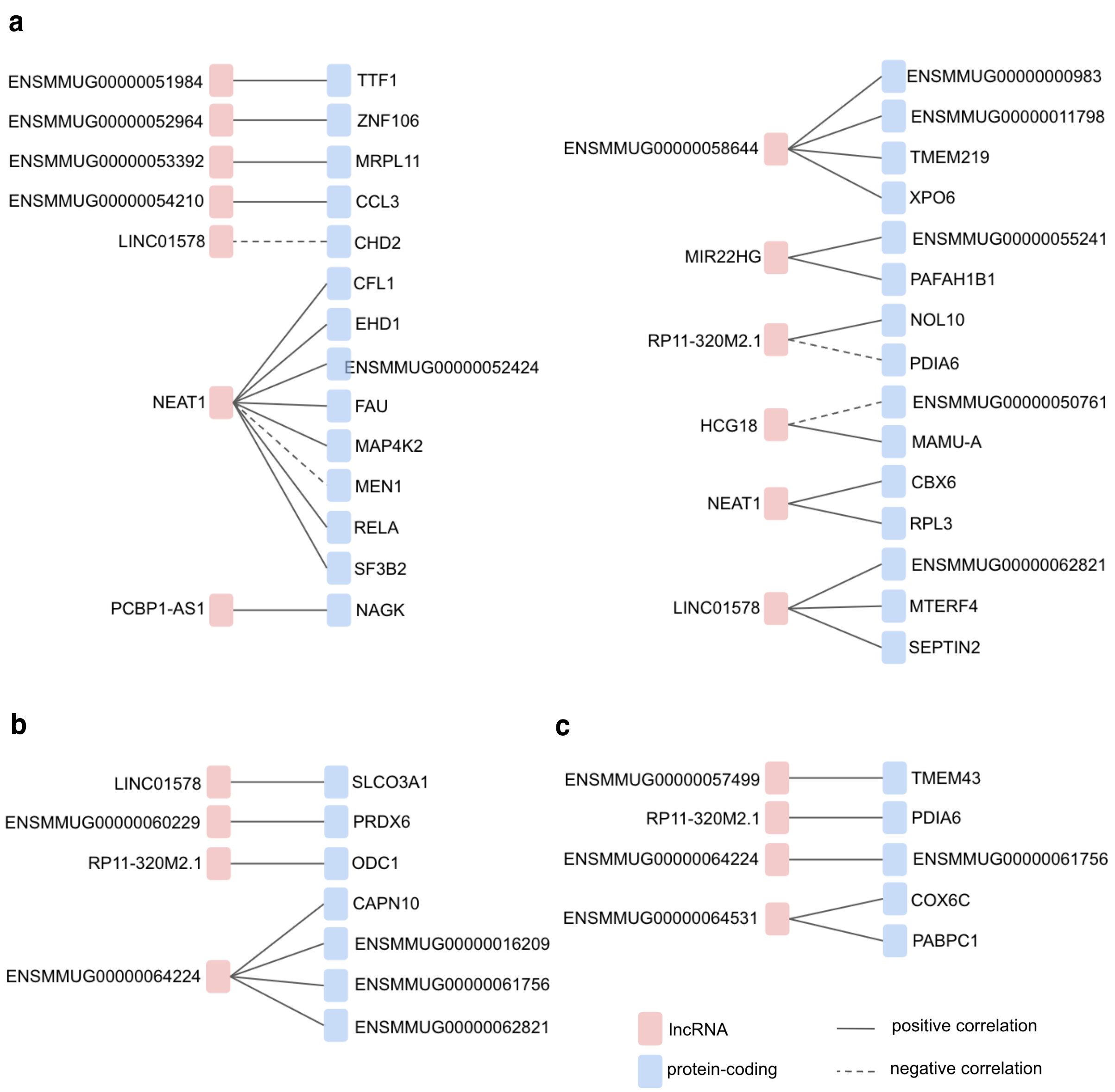

Supplementary Fig. 9. Characterization of *cis*-regulatory activity of lncRNAs. Neighboring lncRNAs (red) and protein-coding genes (blue) DE, co-located and whose expression was significantly correlated (Spearman correlation test, P-value < 0.05) upon EBOV infection *in vivo* in monocytes (**A**), T (**B**), and B (**C**) cells.

Supplementary Figure 10

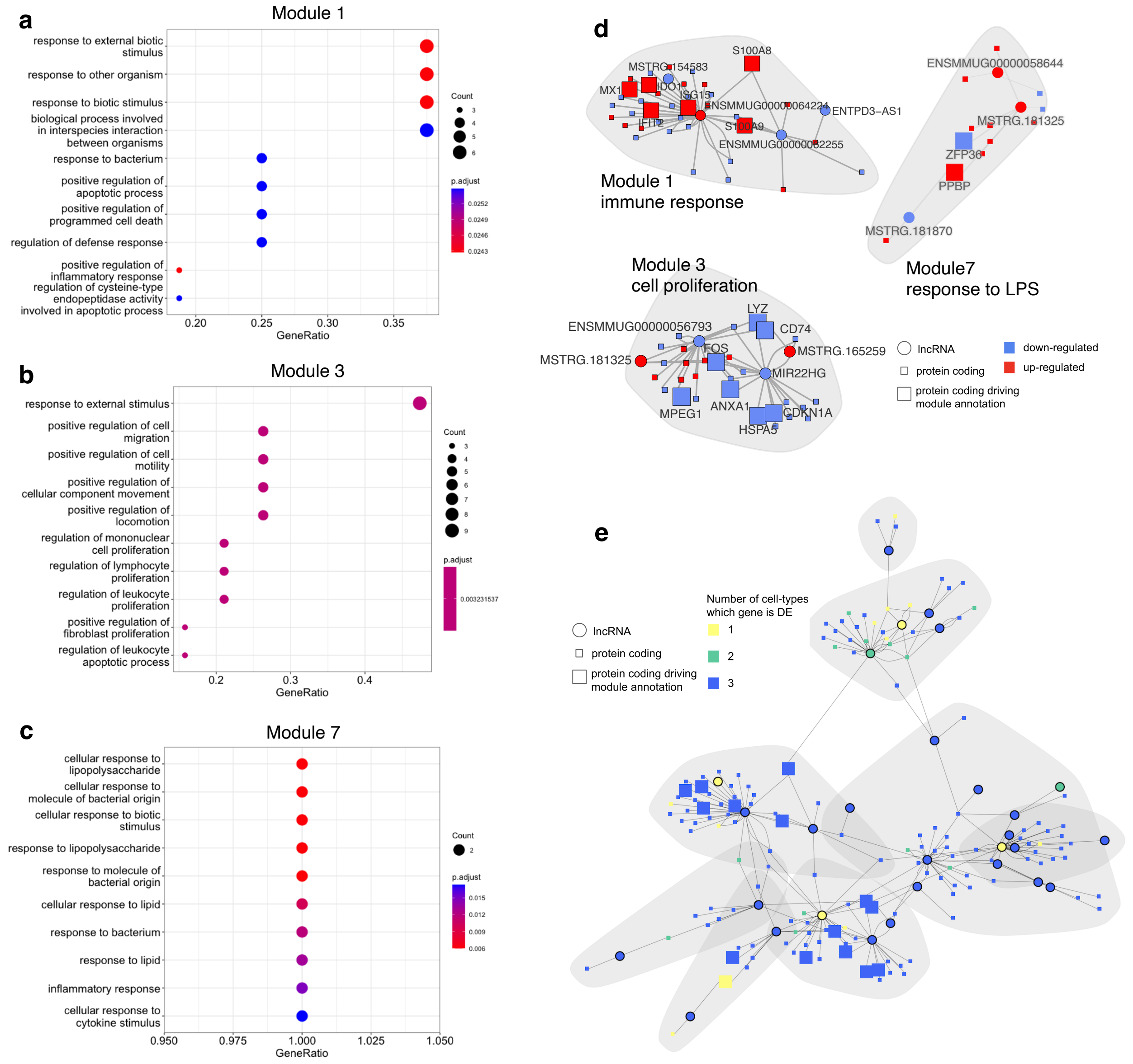

Supplementary Fig. 10. Co-expression network of DE lncRNAs and protein-coding genes upon EBOV infection in monocytes. GO functional enrichment of **(A)** module 1, **(B)** module 3 and **(C)** module 7. **(D)** Zoom-in of the modules of the regulatory network that have significant enrichments. Names of the lncRNAs and protein-coding genes driving the enrichments are displayed. Vertices' colors represent whether a gene is up- or down-regulated upon EBOV infection. Protein-coding genes driving the functional enrichments of each module and lncRNAs are highlighted in larger sizes. **(E)** Regulatory network of DE lncRNAs (circles) and DE protein-coding genes (squares). Vertices' colors represent whether a gene is DE in 1 (yellow), 2 (green) or 3 (blue) cell-types. Protein-coding genes driving the functional enrichments of each module and lncRNAs are highlighted in larger sizes

Supplementary Figure 11

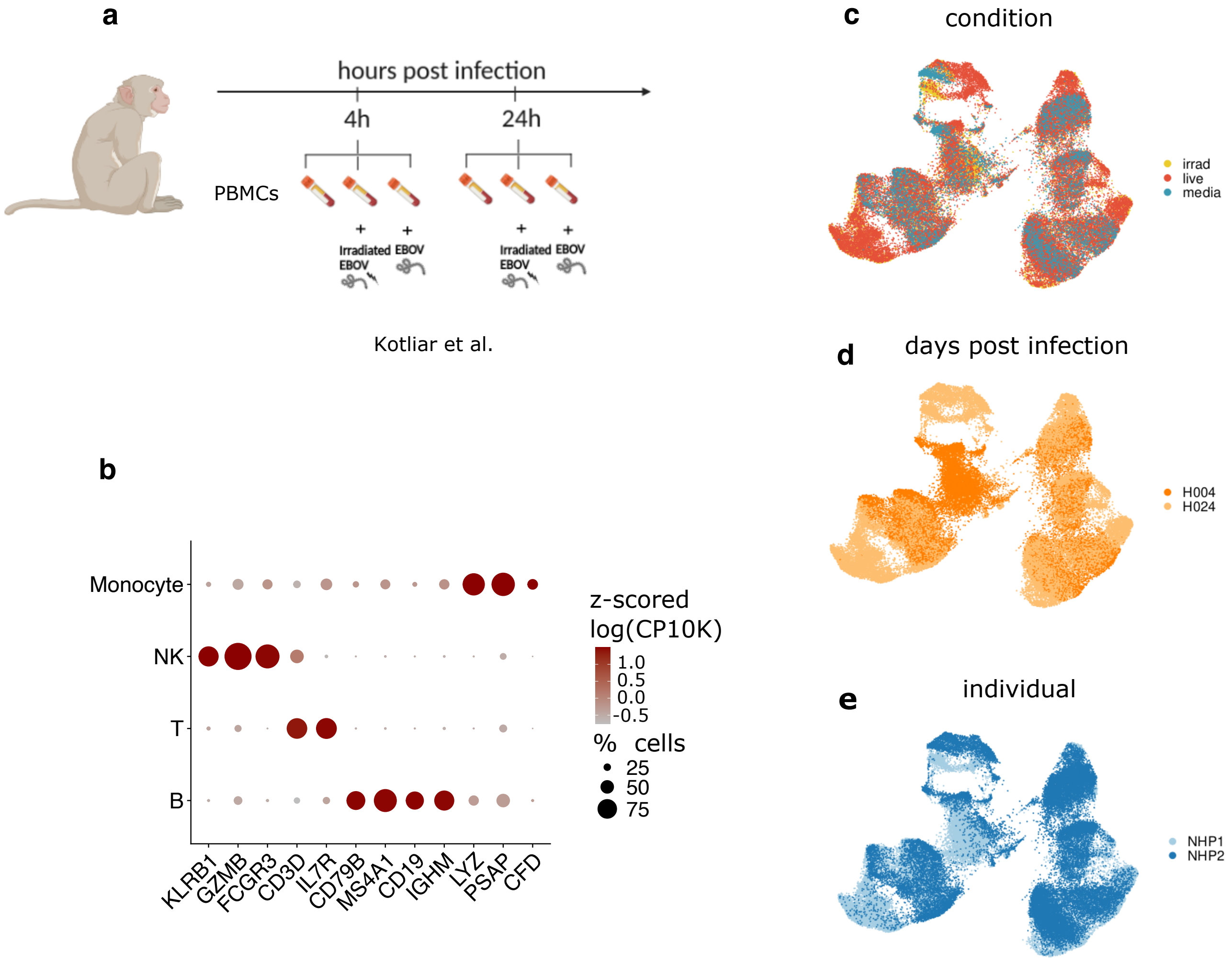

Supplementary Fig. 11. *Ex vivo* dataset processing. **(A)** Schematic overview of the *ex vivo* experiment design. **(B)** Dot plot showing expression of cell-type markers which were used for cell type assignment in the *ex vivo* dataset (see Methods). Dots' colors represent the average expression level of the gene in each cell type. Dots' sizes represent the percentage of cells in which the gene is detected as expressed per cell type. UMAP embedding of cells from the *ex vivo* dataset colored by (**C**) condition , (**D**) sampling hour relative to infection hour, and (**E**) non-human primate.

Supplementary Figure 12

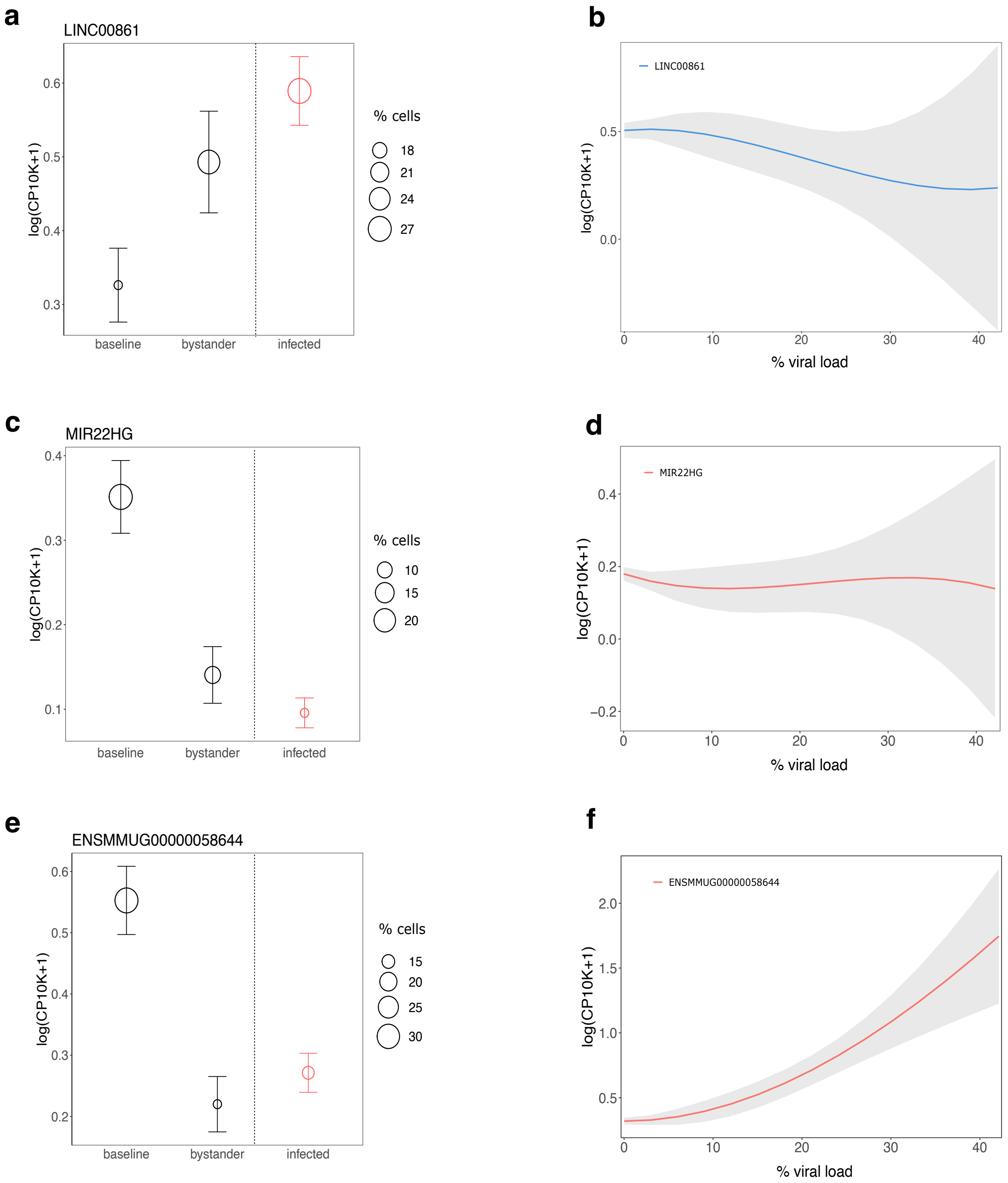

Supplementary Fig. 12. LncRNAs undergo expression changes within infected monocytes. Expression in baseline as well as bystander and infected cells at 24 hours post-infection. Dots' center represent the mean. Error bars indicate the 95% confidence interval around the mean, calculated using the standard error of the mean (SEM). Dots' sizes represent the percentage of cells in which the gene is expressed in each cell group and expression changes with the viral load of **(A-B)** *LINC00861* ,**(C-D)** *MIR22HG* **,** and **(E-F)** *ENSMMUG00000058644* . The shaded area around the smoothed line represents the 95% confidence interval, computed using the loess smoothing method.
