# Supplementary material for: Single-cell profiling of lncRNA expression during Ebola virus infection in rhesus macaques
Source: Nat Commun. 2023 Jun 30;14:3866. doi: 10.1038/s41467-023-39627-7 (PMC10313701; doi:10.1038/s41467-023-39627-7)
Supplement: Supplementary file 3 — Description of Additional Supplementary Files [file 41467_2023_39627_MOESM3_ESM.pdf]

Description of Additional Supplementary Files

File name: **Supplementary Data 1**

Description: All bulk RNA-Seq datasets used in this study.

File name: **Supplementary Data 2**

Description: Differentially expressed lncRNAs, their gene identifier, their annotation origin (annotated vs novel), and, if any, their human ortholog. P-values and logFC are also provided.

File name: **Supplementary Data 3**

Description: Co-expressed modules of differentially expressed lncRNAs and protein-coding genes.

File name: **Supplementary Data 4**

Description: LncRNAs whose expression correlate with viral load within infected cells
