# Supplementary material for: Single-cell profiling of lncRNA expression during Ebola virus infection in rhesus macaques
Source: Nat Commun. 2023 Jun 30;14:3866. doi: 10.1038/s41467-023-39627-7 (PMC10313701; doi:10.1038/s41467-023-39627-7)
Supplement: Supplementary file 8 — Reporting Summary [file 41467_2023_39627_MOESM8_ESM.pdf]

nature portfolio

Corresponding author(s): Lisa E Hensley, Pardis C Sabeti and Marta Melé

Last updated by author(s): May 4, 2023

Reporting Summary

Nature Portfolio wishes to improve the reproducibility of the work that we publish. This form provides structure for consistency and transparency in reporting. For further information on Nature Portfolio policies, see our Editorial Policies and the Editorial Policy Checklist.

Statistics

For all statistical analyses, confirm that the following items are present in the figure legend, table legend, main text, or Methods section.

n/a	Confirmed	
☐	☒	The exact sample size (*n*) for each experimental group/condition, given as a discrete number and unit of measurement
☐	☒	A statement on whether measurements were taken from distinct samples or whether the same sample was measured repeatedly
☐	☒	The statistical test(s) used AND whether they are one- or two-sided<br>*Only common tests should be described solely by name; describe more complex techniques in the Methods section.*
☐	☒	A description of all covariates tested
☐	☒	A description of any assumptions or corrections, such as tests of normality and adjustment for multiple comparisons
☐	☒	A full description of the statistical parameters including central tendency (e.g. means) or other basic estimates (e.g. regression coefficient) AND variation (e.g. standard deviation) or associated estimates of uncertainty (e.g. confidence intervals)
☐	☒	For null hypothesis testing, the test statistic (e.g. *F*, *t*, *r*) with confidence intervals, effect sizes, degrees of freedom and *P* value noted<br>*Give P values as exact values whenever suitable.*
☒	☐	For Bayesian analysis, information on the choice of priors and Markov chain Monte Carlo settings
☒	☐	For hierarchical and complex designs, identification of the appropriate level for tests and full reporting of outcomes
☐	☒	Estimates of effect sizes (e.g. Cohen's *d*, Pearson's *r*), indicating how they were calculated

Our web collection on statistics for biologists contains articles on many of the points above.

Software and code

Policy information about availability of computer code

Data collection	No software was used.
Data analysis	The analysis code is made available at https://github.com/Mele-Lab/2023_SingleCellEbolaLncRNAs_NatComms.<br>R v3.6.2, Hisat v2.1.0, RSeQCc v3.0.0, samtools v1.9, umi_tools v1.0.0, Stringtie v1.3.6, Gffcompare v0.10.6, slncky v1.0, CPAT v3.0.0, CPC v2.0 (http://cpc2.gao-lab.org/data/CPC2-beta.tar.gz), CNIT v2 (http://cnit.noncode.org/CNIT/data/CNIT.tar.gz), Scrublet v.0.2.1, Seurat v3.0,GrnBoost v2.<br>Versions of R packages: MatchIt v4.0.0, MAST v1.12.0, GenomicRanges v1.38.0, clusterProfiler v4.2.0.<br>Docker files are available at: https://github.com/Mele-Lab/2023_SingleCellEbolaLncRNAs_NatComms.<br>Docker images are available at https://hub.docker.com/u/luisas.<br>Nextflow and Singularity were used to run the analysis in a reproducible manner.

For manuscripts utilizing custom algorithms or software that are central to the research but not yet described in published literature, software must be made available to editors and reviewers. We strongly encourage code deposition in a community repository (e.g. GitHub). See the Nature Portfolio guidelines for submitting code & software for further information.

Data

Policy information about availability of data

All manuscripts must include a data availability statement. This statement should provide the following information, where applicable:

- Accession codes, unique identifiers, or web links for publicly available datasets
- A description of any restrictions on data availability
- For clinical datasets or third party data, please ensure that the statement adheres to our policy

The sequencing data generated in this study have been deposited in the NCBI Gene Expression Omnibus (GEO) database under accession code GSE192447 (https://www.ncbi.nlm.nih.gov/geo/query/acc.cgi?acc=GSE192447).

The publicly available whole blood bulk short-read RNA-Seq data from healthy samples and samples infected with Makona Ebola Virus data used in this study are available in the NCBI Gene Expression Omnibus (GEO) database under accession code GSE115785 (https://www.ncbi.nlm.nih.gov/geo/query/acc.cgi?acc=GSE115785).

The single-cell RNA-Seq data used in this study are available in the NCBI Gene Expression Omnibus (GEO) database under accession code GSE158390 (https://www.ncbi.nlm.nih.gov/geo/query/acc.cgi?acc=GSE158390).

The assembly and reference genome of EBOV used in this study are available in the GenBank database under accession code KU182905.1 (https://www.ncbi.nlm.nih.gov/nuccore/KU182905.1).

The assembly and reference genome of Macaca Mulatta used in this study are available in the Ensembl database under accession code Mmul_10 (https://ftp.ensembl.org/pub/release-100/fasta/macaca_mulatta/dna/Macaca_mulatta.Mmul_10.dna.toplevel.fa.gz, https://ftp.ensembl.org/pub/release-100/gtf/macaca_mulatta/Macaca_mulatta.Mmul_10.100.gtf.gz).

The assembly and reference genome of human used in this study are available in the Gencode database under accession code hg38 (https://ftp.ebi.ac.uk/pub/databases/gencode/Gencode_human/release_23/gencode.v23.annotation.gtf.gz, https://ftp.ebi.ac.uk/pub/databases/gencode/Gencode_human/release_23/GRCh38.primary_assembly.genome.fa.gz).

Human research participants

Policy information about studies involving human research participants and Sex and Gender in Research.

Reporting on sex and gender	NA
Population characteristics	NA
Recruitment	NA
Ethics oversight	NA

Note that full information on the approval of the study protocol must also be provided in the manuscript.

Field-specific reporting

Please select the one below that is the best fit for your research. If you are not sure, read the appropriate sections before making your selection.

☒ Life sciences ☐ Behavioural & social sciences ☐ Ecological, evolutionary & environmental sciences

For a reference copy of the document with all sections, see nature.com/documents/nr-reporting-summary-flat.pdf

Life sciences study design

All studies must disclose on these points even when the disclosure is negative.

Sample size

We generated short-read RNA-sequencing data from 13 tissues of not infected (16 samples) and EBOV infected (43 samples) macaques. We further combined this data with publicly available blood RNA-sequencing of not infected (21 samples) and EBOV infected (39 samples) macaques, adding up to a total of 119 samples and almost 4 billion reads. This sample size was enough for us to almost double the current lncRNA rhesus macaque annotation, as reported in the manuscript.

For the single-cell analysis, we used two publicly available single-cell RNA-Seq datasets of Rhesus Macaque peripheral mononuclear cells (PBMCs) infected with EBOV in vivo and ex vivo (see Methods). The in vivo dataset comprised samples from 21 individuals, collected before and at several days post-infection (DPI) with EBOV, and contained 38,067 cells. The ex vivo dataset included PBMCs from healthy macaques,

	either inoculated, irradiated, or incubated with the virus, that were sequenced at 4 or 24 hours post-infection and it contained 56,317 cells. These datasets were previously used by Kotliar et al. and were shown to be sufficient to obtain statistically significant results.<br><br>1.Kotliar, D. et al. Single-Cell Profiling of Ebola Virus Disease In Vivo Reveals Viral and Host Dynamics. Cell 183, 1383 (2020).
Data exclusions	In bulk RNA-Seq data, lowly-expressed genes (log(TPM) > 0.5) were excluded. Single-cell RNA-Seq data was filtered using pre-established thresholds detailed in the manuscript that considered UMIs, number of genes detected and percentage of mitochondrial reads per cell. We also removed doublets using and established software.
Replication	We have used a single-cell healthy human PBMC dataset to replicate our observations
Randomization	This study included the dataset generated by Kotliar et al., where NHPs were randomized into cohorts balancing age, weight, and sex across 7 groups.<br><br>Kotliar, D. et al. Single-Cell Profiling of Ebola Virus Disease In Vivo Reveals Viral and Host Dynamics. Cell 183, 1383 (2020).
Blinding	Blinding is not necessary as in this study the results are obtained with computational analyses that do not require subjective interpretation.

Reporting for specific materials, systems and methods

We require information from authors about some types of materials, experimental systems and methods used in many studies. Here, indicate whether each material, system or method listed is relevant to your study. If you are not sure if a list item applies to your research, read the appropriate section before selecting a response.

Materials & experimental systems

n/a	Involved in the study
☒	☐ Antibodies
☒	☐ Eukaryotic cell lines
☒	☐ Palaeontology and archaeology
☐	☒ Animals and other organisms
☒	☐ Clinical data
☒	☐ Dual use research of concern

Methods

n/a	Involved in the study
☒	☐ ChIP-seq
☒	☐ Flow cytometry
☒	☐ MRI-based neuroimaging

Animals and other research organisms

Policy information about studies involving animals; ARRIVE guidelines recommended for reporting animal research, and Sex and Gender in Research

Laboratory animals	Macaca Mulatta
Wild animals	No wild animals were part of this study.
Reporting on sex	Sex is unnanotated
Field-collected samples	Field-collected samples were not part of this study.
Ethics oversight	The study was performed in accordance with the Guide for the Care and Use of Laboratory Animals of the National Institute of Health, the Office of Animal Welfare, and the US Department of Agriculture (Bennett et al., 2020)

Note that full information on the approval of the study protocol must also be provided in the manuscript.
